# Revising the MELD Score to Address Sex-Bias in Liver Transplant Prioritization for a German Cohort

**DOI:** 10.3390/jpm13060963

**Published:** 2023-06-07

**Authors:** Maria Beatriz Walter Costa, Christiane Gärtner, Maria Schmidt, Thomas Berg, Daniel Seehofer, Thorsten Kaiser

**Affiliations:** 1 Clinical Chemistry and Molecular Diagnostics, Institute for Laboratory Medicine, Leipzig University Medical Center, Paul-List-Straße 13/15, D-04103 Leipzig, Germany; 2 Viral Ecology and Omics, Institute of Biodiversity, Faculty of Biological Sciences, Friedrich Schiller University Jena, Rosalind-Franklin Straße 1, D-07745 Jena, Germany; 3 Academic Department of Laboratory Medicine, Microbiology and Pathobiochemistry, Medical School and University Medical Center East Westphalia-Lippe, Hospital Lippe, Bielefeld University, Röntgenstraße 18, D-32756 Detmold, Germany; 4 Division of Hepatology, Department of Medicine II, Leipzig University Medical Center, Liebigstraße 20, D-04103 Leipzig, Germany; 5 Division of Hepatobiliary Surgery and Visceral Transplant Surgery, Department of Visceral, Transplant, Thoracic and Vascular Surgery, Leipzig University Medical Center, Liebigstraße 20, D-04103 Leipzig, Germany; daniel.seehofer@medizin.uni-leipzig.de

**Keywords:** sex disparity, gender bias, liver transplantation, MELD, MELD 3.0, sex dimorphism

## Abstract

(1) Background: Prioritization of patients for liver transplantation in Germany relies on the MELD (model for end-stage liver disease) scoring system that does not consider the patient’s sex. Many studies have shown that women are disadvantaged by the MELD score. Using a large patient cohort from a German liver transplant centre, we investigated options to reduce gender inequality in the patient prioritization for liver transplantation. (2) Methods: We calculated female-as-male MELD scores in our cohort by substituting the serum creatinine of a female patient with that of their male equivalent to test for the fairness of the scores. We investigated the effects of the female-as-male scores compared to the original MELD score of 1759 patients listed for liver transplantation. (3) Results: Serum creatinine sex correction (female-as-male) for MELD scores added up to 5.4 points in females, while the median changed by +1.6 points for females. We identified 72 females with an original MELD score < 20, for whom the adjusted female-as-male MELD score would be >20, thus giving them a better chance to receive a liver transplant. (4) Conclusions: Mathematical conversion of female to male creatinine concentrations identified disadvantages in liver transplantation prioritization for females and ascertained MELD 3.0 as having high potential to compensate for these inequalities.

## 1. Introduction

Due to an imbalance between the number of organ donors and recipients, many patients are waiting for a donor organ in Germany [1]. In the Eurotransplant region, around 1600 livers are available annually for transplant from deceased patients. Prioritizing patients for liver transplantation in the Eurotransplant region relies on the MELD (model for end-stage liver disease) scoring system that stratifies recipients on the waiting list based on their disease severity and 3-month probability of death [2]. The original MELD scoring system is based on the serum creatinine (SCr), bilirubin, and INR (international normalized ratio, standardized prothrombin time). However, the score does not consider the sex of the patient and was generated for a US cohort and later introduced in the Eurotransplant region in 2003 [3].

Due to physiological differences, median SCr levels are higher in males than in females [4]. Among other things, this causes a biased MELD scoring between sexes, with a tendency for lower scores in females, even in otherwise similar health conditions [5]. Thus, to address this disadvantage in the traditional MELD scoring system, different adjustments to the MELD score have been proposed, such as MELD 3.0 and MELDNa-Shift [6,7]. MELD 3.0 adds 1.4 points to the MELD score if patients are female, whereas MELDNa-Shift adds one point to specific MELD values of female patients.

We aimed to investigate sex bias in the MELD score calculations for a cohort in Germany. We first compared the MELD score assigned to a female patient with their female-to-male score. This is possible because blood SCr concentrations can be converted to estimated glomerular filtration rate (eGFR) and vice versa using the established CKD-EPI equation, which includes sex and age [8]. Next, we calculated the adjusted MELD scores for each female patient, which would consider the sex of the patient. Finally, we compared the different scores and evaluated the extent of sex bias reduction.

## 2. Methods

The dataset used in this project consists of the MELD scores and related parameters of adult patients (age ≥ 18 years) at the University of Leipzig Medical Center who were listed for their first liver transplantation between 2012 and 2020. The MELD scores were measured using the lab-MELD validation and reporting system [9] at the Institute of Laboratory Medicine of the University of Leipzig Medical Center. Bilirubin was measured photometrically using the “Bilirubin Total Gen.3” test kit on Cobas8000 c701. We used colorimetric enzymatic assays to obtain SCr levels (CREPs2/Creatinine plus ver.2) and ion-sensitive electrode Gen2 to measure sodium levels on Cobas8000 (Roche Diagnostics). For INR calculation, prothrombin time was measured on ACL as coagulometry using the “HaemosIL Thrombin Time” test kit. The eGFR was calculated using the CKD-EPI equation [10].

Using a validated custom R script, we computed the MELD score for the male counterpart of a female patient by substituting the female patient’s SCr with their male equivalent. We calculated the female-to-male SCr value as the SCr with the same age but a male SCr value after conversion to eGFR and back. For a detailed description of the calculations, see [8] and Appendix A.1.

For subsequent comparisons, MELD 3.0 [6], MELDNa [11], and MELDNa-Shift [7] scores were calculated using their original equations and with the female-to-male SCr. MELD scores > 40 were set to 40. Equations used for adjustments can also be found in Appendix A.2. The median laboratory MELD score for transplantation in Germany is approximately 20, according to Umgelter et al. [12] and Ritschl et al. [13]. Therefore, we assigned 20 as a threshold value for transplantation to calculate the number of female patients who would have increased access to liver transplantation when using the female-to-male SCr value for the calculation of the MELD scores.

For comparison of non-normal distributions, we used the non-parametric Mann–Whitney U test. For effect size calculations of non-normal distributions, we used the Vargha and Delaney measure (R companion package [14]). Results are presented as the median and interquartile ranges unless otherwise specified. We performed the analysis and calculations with R 4.1.3 [15], plyr [16], and dplyr [17], and created the plots using ggplot2 [18].

## 3. Results

Our cohort consists of 1759 patients, of which 650 (37%) are female. Table 1 gives an overview of all the baseline characteristics of the patients. Age, SCr, INR, and eGFR are significantly different between sexes (p<0.05) but with small effect sizes. Age and INR are significantly different between males and females (p<0.05) but with no effect size.

**Figure 1:**
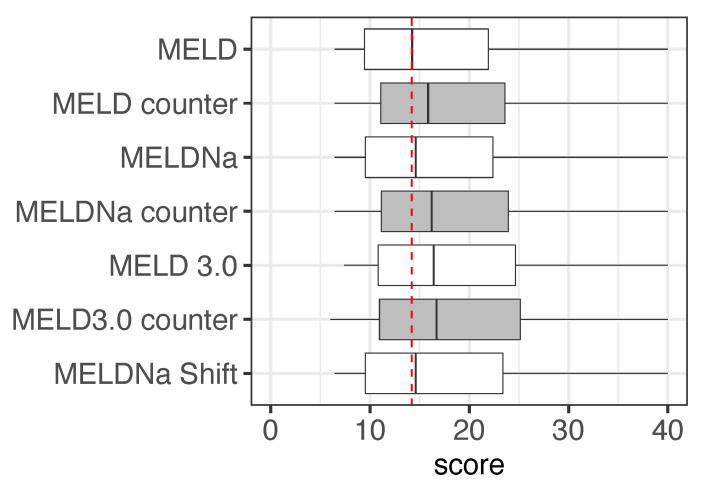
Comparison of the original and adjusted MELD scoring systems for female patients. We only included the original MELD scores ≥ 15. Scores were calculated for the actual MELDNa-Shift and the actual and female-to-male counterparts of MELD, MELDNa, and MELD 3.0. The red dashed line shows the median of the original MELD score.

MELD and MELDNa scores resulted in considerable changes to the transplantation prioritization. Calculating the MELD score using the female-to-male SCr values added a median score of 2.2 and up to 5.4 points to female MELD and MELDNa scores (Figure 1). MELD and MELDNa scores calculated with the original SCr and the female-to-male SCr values differed significantly for female patients (*p* < 0.05). In contrast, for the MELD 3.0 scores of female patients, only a median of 0.3 points were added when using the female-to-male SCr values. In addition, this change was not statistically significant.

To gain deeper insight into the possible disadvantages for females concerning access to liver transplantation, we calculated the number of female patients with an original MELD, MELD 3.0, or MELDNa score ≥ 15 and <20, who would score > 20 when using the female-to-male SCr values for calculation.

**Figure 2:**
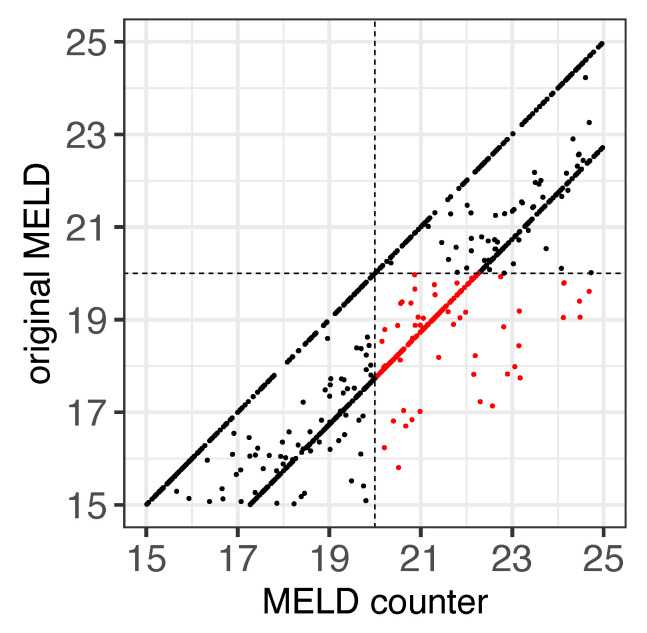
Comparison of the MELD score for females and their male counterparts for a threshold of 20. Original MELD scores are shown on the *y*-axis, and the female-to-male counterpart MELD scores on the *x*-axis. Only original MELD measurements that were ≥15 and <25 are shown. Highlighted in red are the scores that are upgraded to the transplant category, considering a threshold of 20 (horizontal and vertical dashed lines). Note that the MELD scores are upgraded to the transplant category region for females when their female-to-male counterpart is considered in the score calculation.

**Figure 3:**
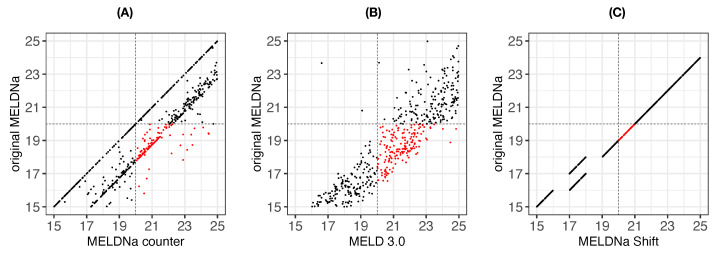
Original versus the female-to-male counterpart MELDNa scores and the adjusted MELDNa scores for a threshold of 20. The *y*-axis shows the female patients’ original MELDNa scores that are ≥15 and <25. (**A**) Females’ original MELDNa scores (*y*-axis) versus female-to-male counterpart MELDNa scores (*x*-axis). (**B**) Females’ original MELDNa scores (*y*-axis) versus MELD 3.0 scores (*x*-axis). (**C**) Females’ original MELDNa scores (*y*-axis) versus MELDNa-Shift scores (*x*-axis). Highlighted in red are the scores that are upgraded to the transplant category, considering a threshold of 20 (horizontal and vertical dashed lines). Note that the MELD scores are upgraded to the transplant category region for females when considering their female-to-male counterpart in the score calculation.

Importantly, we observed 72 female patients, reflected by 165 single MELD scores < 20, who would be assigned a MELD score ≥ 20 if they were male (Figure 2, red dots). These patients correspond to 31.3% of the female patients with an original MELD score ≥ 15 and <20. In contrast, no female patient with an original MELD ≥ 20 would receive a MELD < 20 if they were male (Figure 2, top left quarter). We observed similar results for the MELDNa score (Figure 3A). This shows the relevance of the bias not only in the Eurotransplant region, where the MELD score is used for listing patients for a liver allocation, but also in the UNOS region, where the MELDNa score is used to prioritize patients for a liver allocation.

Additionally, we tested the potential differences between the original MELDNa scores and their adjustments, MELD 3.0 and MELDNa-Shift scores, in female patients. In this case, we used MELDNa instead of the MELD score as the gold standard for comparison because both MELDNa-Shift and MELD 3.0 are adjustments of MELDNa. Moreover, MELD and MELDNa cannot be directly compared due to differences in the input parameters.

Using the MELD 3.0 adjustment, 84 patients received a score ≥ 20 (Figure 3B). Applying the MELDNa-Shift adjustment to the females’ MELDNa scores resulted in 51 female patients with MELDNa scores between 15 and 20 points receiving MELDNa-Shift scores ≥ 20 (Figure 3C).

**Figure 4:**
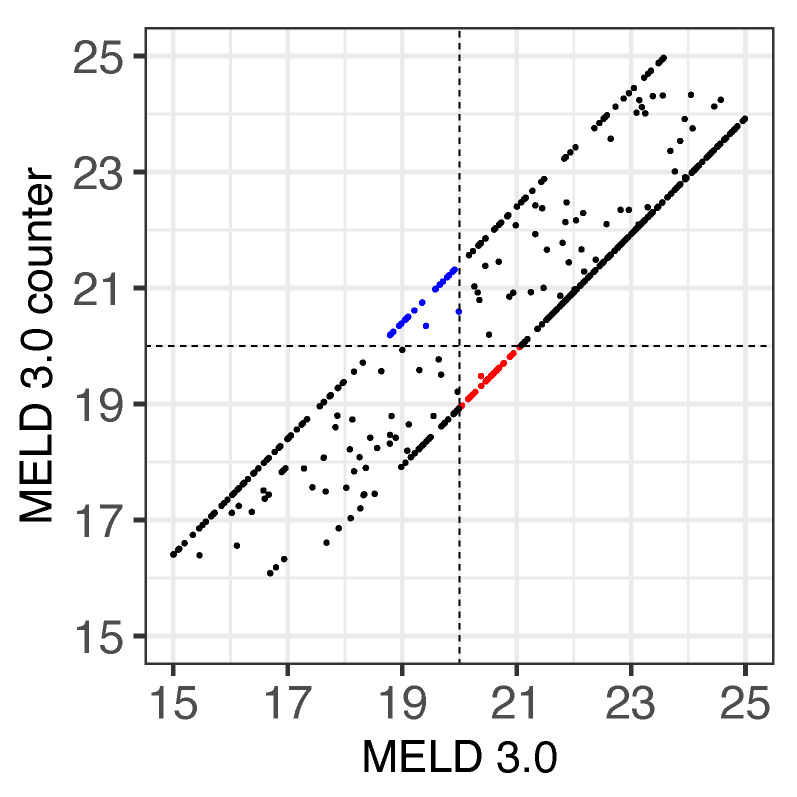
Comparison of the MELD scores for females and their female-to-male counterparts for a threshold of 20. Dots represent the female-to-male counterpart MELD 3.0 (*y*-axis) and the original MELD 3.0 scores (*x*-axis). Only measurements with MELD 3.0 scores ≥ 15 and <25 were included. Highlighted in red are the scores that are upgraded to the transplant category, considering a threshold of 20 (horizontal and vertical dashed lines), whereas blue dots indicate a downgrade.

Furthermore, we compared the original MELD 3.0 scores of females with the corresponding female-to-male MELD 3.0 scores (Figure 4) to test whether the MELD 3.0 adjustment could compensate for the sex bias (Figure 4). While 41 female patients received a score ≥ 20 with their female-to-male MELD 3.0 score (Figure 4, red dots), 23 received an female-to-male score < 20, even though they had an original MELD 3.0 score ≥ 20 (Figure 4, blue dots).

## 4. Discussion

Converting women’s creatinine results using the accepted CKD-EPI-GFR equation provides a new and objective approach to prevent inequalities in organ transplantation prioritization. For the 242 female patients in our cohort who had an original MELD score between 15 and 20, 31.3% would be upgraded to a score larger than 20 if the female-to-male SCr values are considered for calculations. These patients might have been eligible for a liver transplant earlier. This shows a considerable bias against female patients in our cohort from a German university hospital. The risk for women to be assigned lower MELD scores despite having the same disease severity and equally poor kidney function has been extensively described in various cohorts from Brazil [19], Europe [20], and the UNOS region [21].

A lower MELD score directly translates to decreased access to liver transplantation. One reason for this disparity is a lower median muscle mass in females compared with males. Different MELD score adjustments that aim to remove the bias between males and females in the MELD score calculation have already been suggested. We analysed MELDNa-Shift, which adds points to the MELDNa scores for females [7], and MELD 3.0 [6], which adds 1.4 points to female MELDNa scores and adjusts other MELD parameters. Similar to the outlined findings in [7], MELDNa-Shift upgraded the scores of 51 female patients > 20 and partly corrected the sex disparity in our cohort.

Importantly, one should keep in mind that prioritization for a liver allocation in the Eurotransplant region relies on the MELD score. In contrast, MELD 3.0 and MELDNa-Shift are adjustments for the MELDNa equation used in the UNOS region. Our results agree with the findings of Kim et al. [6] in that the chance for transplantation was higher among females when using MELD 3.0 compared to MELDNa or MELD.

Our results are in good agreement with the findings of Sealock and colleagues, who also found a disadvantage for women when using the MELD score for a US cohort [22]. In their study, the disadvantage against women could be partly resolved in simulated data with an adjustment of MELDNa calculation, thereby lowering the overall death rate and increasing access to transplantation for women.

Locke and colleagues [23] found that the deceased access to donor liver transplantation for females is 14.6% less likely than males. They pointed out that besides SCr levels, anthropometric and liver measurements also play a role in the observed access bias, as shown by the influence of height on the decreased access to liver transplantation in [24]. As the median height of women is smaller than that of men, they face a disadvantage. Furthermore, several publications have discussed the influence of the post-menopausal hormonal status on the decreased access of women to liver transplantation. Oestrogens have a potentially protective role in the progression of liver fibrosis, which can lead to liver cirrhosis and the need for liver transplantation [25]. This protection is lost during menopause [25]. In addition to the physiological factors mentioned above, social factors also play a significant role, such as a generally reduced evaluation and consideration for liver transplantation among women [26].

Generally, one should keep the lower muscle mass and changed hormonal status in mind when evaluating patients with liver cirrhosis. This issue raises the question of whether SCr as well as the MELD scoring system are in fact helpful for evaluating patients with liver cirrhosis, regardless of the patient’s sex. O’Leary and Bajaj [27] also pointed out that MELD adjustments should rely on objective muscle mass quantification rather than sex or height correction.

### Perspectives and Significance

In summary, we confirmed a sex bias similar to that observed in cohorts from different countries [19,20,21] at our medical centre in Germany. Although this problem has been known for years, an adequate solution is still lacking. Adjustments to the MELD score, such as MELD 3.0 [6] and MELDNa-Shift [7], developed based on US cohorts have been proposed. We searched for the best possible alternatives for our patients. MELD 3.0 has the potential to correct the sex bias in our cohort. We also presented an additional alternative MELD score for females using the SCr values of their female-to-male counterparts for the calculation. Although it is not yet fully clear how to increase female access to liver transplantation in Germany, this study provides additional information about the MELD score bias against females and a possible remedy, creating a more suitable base for clinicians’ decision making regarding prioritization for liver transplantation.

## 5. Conclusions

Our results underscore the urgent need for sex correction in the MELD score calculations. The use of MELD 3.0 score or the more laborious incorporation of SCr values adjusted for sex may partly resolve this issue. Our results and the sex-specific interpretation of laboratory diagnostics should be the subject of further research, which should include clinical endpoint evaluation.

## Figures and Tables

**Table 1:** Basic statistics of the studied cohort. Percentages are given in round brackets. Continuous values are given as medians with interquartile ranges in square brackets. SCr: Serum creatinine.

	Males	Females	All
Measurements (n, %)	5541 (63.5)	3185 (36.5)	8726 (100)
Patients (n, %)	1109 (63.0)	650 (37.0)	1759 (100)
Age (yrs) ${}^{*}$	57.4 [51.1; 63.3]	56.6 [49.8; 55.3]	57.2 [50.7; 63.2]
SCr (μmol/L) ${}^{*}$${}^{+}$	89.0 [73.0; 123.0]	81.0 [63.0; 115.0]	86.0 [69.0; 120.0]
Bilirubin (μmol/L)	32.0 [17.0; 67.4]	34.4 [15.8; 93.9]	32.9 [16.6; 73.7]
INR ${}^{*}$	1.3 [1.2; 1.6]	1.4 [1.2; 1.7]	1.3 [1.2; 1.6]
eGFR (CKD-EPI) (mL/min/1.73 m^2^) ${}^{*}$${}^{+}$	83.0 [55.7; 98.6]	70.0 [45.4; 94.6]	78.2 [50.7; 97.5]
MELD score	13.8 [9.9; 20.2]	14.3 [9.5; 21.9]	14.0 [9.7; 20.7]

${}^{*}$ statistically different distributions (*p*-value < 0.05). ${}^{+}$ small effect (Vargha and Delaney).

## Data Availability

Our data are available upon request. Although data are pseudonymized, details such as the combination of sex, age, diagnoses, and period the of assessment at ULMC could potentially be used to identify individual cases and patients. Therefore, in accordance with the General Data Protection Regulation, we are restrained from the public release of our data set. When administrative and legal requirements are met, trusted research institutions may request data access by contacting the current director of the Institute of Laboratory Medicine, Clinical Chemistry and Molecular Diagnostics at the University of Leipzig (MB-sek-ilm@medizin.uni-leipzig.de) or the AMPEL project directly (MB-ilm-ampel@medizin.uni-leipzig.de).

## Abbreviations

The following abbreviations are used in this manuscript:

MELD	Model for end-stage liver disease
CKD-EPI	Chronic kidney disease epidemiology collaboration
eGFR	Estimated glomerular filtration rate
SCr	Serum creatinine

## Appendix A

### Appendix A.1. Equations (A1)–(A4): Calculation of the Opposite Sex Counterpart Serum Creatinine Values

The calculation of eGFR (estimated glomerular filtration rate) from serum creatinine (SCr) values requires the use of two different CKD-EPI (chronic kidney disease epidemiology collaboration, [10]) equations per gender, depending on whether SCr is above or below/equal to a specific limit of 0.7 mg/dL for females and 0.9 mg/dL for males. Thus, the back-calculation of the opposite sex counterpart SCr value from eGFR also yields two equations per sex as well as sex-adapted changes to the parameters in the equations. Details of the methodology can be found in [8].

**Male counterpart SCr for females:**

Above the sex-specific limit value:

(A1) $$SCr\left(\mathrm{mg}/\mathrm{dL}\right)=e^{ln(eGFR/141/(0.993^{Age}/-1.209)}*88.42*0.9$$

Below/equal to the sex-specific limit value:

(A2) $$SCr\left(\mathrm{mg}/\mathrm{dL}\right)=e^{ln(eGFR/141/(0.993^{Age}/-0.411)}*88.42*0.9$$

**Female counterpart SCr for males:**

Above the sex-specific limit value:

(A3) $$SCr\left(\mathrm{mg}/\mathrm{dL}\right)=e^{ln(eGFR/143.538/(0.993^{Age}/-1.209)}*88.42*0.7$$

Below/equal to the sex-specific limit value:

(A4) $$SCr\left(\mathrm{mg}/\mathrm{dL}\right)=e^{ln(eGFR/143.5338/(0.993^{Age}/-0.329)}*88.42*0.7$$

### Appendix A.2. Equation (A5): Calculation of MELD 3.0 without Albumin

The equation to calculate MELD 3.0 without albumin follows the methodology outlined in [6].

(A5) $$\begin{array}{cc} \mathrm{MELD}\;3.0\;\mathrm{no}\;\mathrm{albumin}=\; & 1.40\;(\mathrm{if}\;\mathrm{female})+\left[4.85\times log_{e}\left(bilirubin\right)\right]+\left[0.88\times \left(137-Na\right)\right]-\\ \; & \left[0.25\times \left(137-Na\right)\times log_{e}\left(bilirubin\right)\right]+\left[9.66\times log_{e}\left(INR\right)\right]+\\ \; & [10.47\times log_{e}\left(creatinine\right)]+6 \end{array}$$

Bilirubin (mg/dL), serum creatinine (mg/dL), and INR (international normalized ratio) values <1 were set to 1. The lower and upper reference limits for sodium were 125 mmol/L and 137 mmol/L, respectively. Thus, values below or above these limits were set to 125 mmol/L or 137 mmol/L, respectively.
